# Increased Hyaluronan Levels in HABP1/p32/gC1qR Overexpressing HepG2 Cells Inhibit Autophagic Vacuolation Regulating Tumor Potency

**DOI:** 10.1371/journal.pone.0103208

**Published:** 2014-07-25

**Authors:** Paramita Saha, Ilora Ghosh, Kasturi Datta

**Affiliations:** Biochemistry and Toxicology Laboratory, School of Environmental Sciences, Jawaharlal Nehru University, New Delhi, India; Rajiv Gandhi Centre for Biotechnology, India

## Abstract

Tumor growth and development is influenced by its microenvironment. A major extracellular matrix molecule involved in cancer progression is hyaluronan (HA). Hyaluronan and expression of a number of hyaladherin family proteins are dramatically increased in many cancer malignancies. One such hyaladherin, hyaluronan-binding protein 1 (HABP1/p32/gC1qR) has been considered to be a biomarker for tumor progression. Interestingly, overexpression of HABP1 in fibroblast has been shown to increase autophagy via generation of excess reactive oxygen species (ROS) and depletion of HA leading to apoptosis. Cancerous cells are often found to exhibit decreased rate of proteolysis/autophagy in comparison to their normal counterparts. To determine if HABP1 levels alter tumorigenicity of cancerous cells, HepR21, the stable transfectant overexpressing HABP1 in HepG2 cell line was derived. HepR21 has been shown to have increased proliferation rate than HepG2, intracellular HA cable formation and enhanced tumor potency without any significant alteration of intracellular ROS. In this paper we have observed that HepR21 cells containing higher endogenous HA levels, have downregulated expression of the autophagic marker, MAP-LC3, consistent with unaltered levels of endogenous ROS. In fact, HepR21 cells seem to have significant resistance to exogenous ROS stimuli and glutathione depletion. HepR21 cells were also found to be more resilient to nutrient starvation in comparison to its parent cell line. Decline in intracellular HA levels and HA cables in HepR21 cells upon treatment with HAS inhibitor (4-MU), induced a surge in ROS levels leading to increased expression of MAP-LC3 and tumor suppressors Beclin 1 and PTEN. This suggests the importance of HABP1 induced HA cable formation in enhancing tumor potency by maintaining the oxidant levels and subsequent autophagic vacuolation.

## Introduction

Extracellular matrix (ECM) remodeling is one of the prime factors for tumor malignancy and metastatic progression [1]. One of the key components of ECM is Hyaluronan (HA), the only non-sulfated glycosaminoglycan, found in the extracellular and pericellular space. HA has been reported to be dramatically increased in many malignancies. HA rich matrix is associated with different hallmarks of tumor pathobiology like anchorage independent growth, migration, angiogenesis, suppression of apoptosis and metastasis [2]. Altered HA synthesis in tumor cells by HAS activity accelerates tumor growth through the recruitment of HA rich stromal cells and vasculature aided by factors secreted by tumor cells themselves [3], [4]. Consequently, HA synthase inhibitor, 4-MU has been reported to act as anti-tumor agent leading to decreased HA level, growth arrest and apoptosis [5], [6]. Although high molecular weight HA has been observed to enhance cellular proliferation, HA oligosaccharides inhibit anchorage independent growth in tumor cells by suppressing the PI3 Kinase/Akt survival pathway by stimulating expression of the tumor suppressor PTEN [7]. Interestingly, in rat mesengial cells HA cables colocalized with autophagic marker MAP-LC3 under hyperglycemic condition although the significance remains unclear [8], [9]. The process of autophagy is considered to be highly dynamic for tumorigenesis. Not surprisingly a number of molecular factors regulating autophagy also act as tumor suppressors such as Beclin 1, p53, PTEN and p19ARF. Activation of autophagy might help cancer cells survive for extended periods of nutrient deprivation or hypoxic condition [10], [11] and provide an escape route from metabolic stress at later stages of cancer. Contrary to this, several other reports suggest that a decline in cellular proteolysis in numerous cancerous cells [12], [13] results from downregulation of several autophagic markers and modulators like Beclin 1, PTEN and DRAM (a lysosomal protein activated by the tumor suppressor p53) at either transcript or protein level. Overexpression of several such modulators has been found to be instrumental enough to bring a decline in tumorigenicity levels or induce autophagic death in cancerous cells [14]–[21]. Autophagy in 293T cells is induced by the short mitochondrial form (smARF) of p19ARF. The role of short-lived smARF as autophagy inducer is regulated by physical binding with hyaluronan-binding protein 1 (HABP1/p32/gC1qR) and its subsequent translocation to mitochondria [22], [23].

Differential expression of HABP1/p32/gC1qR in skin papilloma [24] and in various adenocarcinomas [25] has been observed. This suggests a probable role of HABP1 in tumor metastasis. HABP1/p32/gC1qR has also been recognized as a receptor for the tumor homing peptide Lyp1 which specifically recognizes an epitope in tumor lymphatics and tumor cells in certain cancers [26]. Knocking down HABP1 in cancer cells makes them less tumorigenic [27]. Interestingly, constitutive overexpression of HABP1 in fibroblasts has been reported to lead to its mitochondrial translocation, induction of autophagy, along with depletion and depolymerization of HA, and subsequent apoptosis as a consequence of excess ROS generation [28], [29]. However, our recent report shows that upon stable transfection of HABP1 in hepatocarcinoma cell line HepG2, having high intracellular antioxidant levels [30], [31], induces increased cellular proliferation, HA synthesis, and HA cable formation along with increased colony forming ability in soft agar assay [32]. This stable HepG2 transfectant developed in our laboratory and termed as HepR21 displayed cell proliferation by upregulation of cyclin D1 in an AKT-dependent pathway, instead of growth retardation; all leading to increased tumor potency [32]. Using silk-fibroin based three dimensional culture system, we confirmed the increased tumor potency of this HABP1 overexpressing HepG2 cell line (HepR21). Reduction in tumor marker was consistently seen upon HA depletion via HAS inhibition [33]. Existing literature indicates downregulation of the autophagic machinery in aggressive cancerous cells. Association of HABP1 with autophagy instigated us to study the correlation of elevated HA level upon overexpression of HABP1 and tumor potency in ROS insensitive cell line HepR21. In this paper we show that HepR21 cells have downregulated expression of autophagic marker MAP-LC3. Intracellular ROS levels remain unchanged in HepR21 cells and in fact they seem to gain significant resistance to exogenous agents capable of elevating ROS levels compared to HepG2 cells. HepR21 cells were also found to be more resilient to nutrient starvation in comparison to its parent cell line. Reduction in intracellular HA levels and HA cables in HepR21 cells upon treatment with HAS inhibitor (4-MU), induced a surge in ROS levels leading to increased expression of autophagy marker MAP-LC3-II and tumor suppressors Beclin 1 and PTEN.

## Materials and Methods

### Reagents

Dulbecco's modified Eagle's medium (DMEM), Fetal bovine serum and all antibiotics were from Sigma Aldrich Chemicals Pvt. Ltd. (USA). Primary and secondary antibodies were obtained from Santa Cruz Biotechnology Inc. (USA), Sigma Aldrich Chemicals Pvt. Ltd. (USA), Abcam Inc (USA) and Cell Signaling Technologies (USA). All chemicals were procured from Sigma Aldrich Chemicals Pvt. Ltd. (USA) unless otherwise specified. Antibody to HABP1/p32/gC1qR was generated in our laboratory as previously described [34]. Alexa Fluor 488 and 546, Streptavidin Alexa Fluor 430 and 2'7'-dichlorodihydrofluorescein diacetate (H_2_DCFDA) were from Molecular Probes Inc. (Eugene, OR). Low molecular weight marker was obtained from Amersham, UK. Cell culture plastic wares were obtained from Corning-Costar Inc. (Corning, NY, USA). 0.22 µM membrane filters for filtering media and PVDF membranes were obtained from Millipore (MA, USA). Filteration unit and isopropanol cryobox were purchased from Nalgene (Nalge Nunc International Corporation, Rochester, NY, USA). Water used for preparing media and reagents was either autoclaved triple distilled (distilled in our laboratory) or autoclaved Milli Q (obtained from water purification system, Millipore, MA, USA).

### Maintenance of Cell Line

All cell lines were cultured in High Glucose DMEM (Dulbeco's Modified Eagle's Medium), containing 4.5 gm/L glucose. Media was supplemented with 10% FCS, 100 µg/ml streptomycin and 50 µg/ml fungizone in tissue culture flasks and dishes. The cultures were grown at 37°C in a humid atmosphere with 5% CO_2_ and 95% air. The cells were routinely maintained in monolayer culture and detached from the plastic surfaces of tissue culture wares by trypsinization (trypsin-EDTA treatment: 0.25% trypsin and 2 mM EDTA in 0.01 M PBS, pH 7.2) for regular sub-culturing.

### Cell Treatments

HepG2 and HepR21 cells were grown in complete media for 48 h and then treated with varying concentrations (0, 50, 100, 250 and 500 µM) of H_2_O_2_ for 1 h and subjected to ROS assay and cell survivability assay according to the process mentioned below.

HepG2 and HepR21 cells grown for 24 h were treated with increasing concentrations (0, 0.25, 0.50, 1 and 10 mM) of buthionine sulfoximine (BSO), a synthetic amino acid which irreversibly inhibits gamma-glutamylcysteine synthetase, for 24 h and then subjected to ROS assay according to protocol mentioned.

For nutrient deprivation in HepG2 and HepR21, cells were treated with Earle's balanced salt solution (EBSS), an amino acid deficient media, already reported to generate autophagic vacuoles in HepG2 [35]. Cells were initially grown in complete media and treated for 6, 12, 24 and 36 h, keeping the total period of growth to be same (48 h) in each case. One set of cells were kept as control and grown for the same time period.

4-methyl umbelliferone (4-MU) has been reported to lead to HA synthesis by depleting the cellular UDP-glucoronic acid and downregulating HAS2 and HAS3 [5]. HepG2 and the higher HA containing HepR21 cells, were grown and exposed to different concentrations of 4-MU (0.25, 0.50, 1 and 2 mM) for either 6 or 12 hours as per the experiment along with a set of untreated cells acting as control. The total period of growth was kept same (48 h) in each case.

### Detection of Autophagic vacuoles with Monodansyl Cadaverin (MDC)

The development of autophagic vacuoles was studied at various growth periods by staining cells with 0.05 mM MDC as reported earlier [28]. MDC specifically binds to Phosphoethanolamine (PE) in the autophagic membrane [36]. Culture media after specified period of growth was discarded and washed with PBS (pH 7.2). Cells were then incubated with 0.05 mM MDC in PBS at 37°C for 10 minutes. After incubation cells were washed 4 times with PBS and immediately analyzed by fluorescence microscopy [Excitation wavelength = 335 nm; Emission wavelength  = 525 nm].

### Immunoblot analysis

The protein samples electrophoresed by SDS-PAGE were electro-blotted on PVDF membrane by applying 0.8 mA/hour current in a wet-transfer unit. Following transfer the membrane was blocked with 5% non-fat dry milk in PBS for 2 hours at room temperature The PVDF membrane was then washed three times with PBST (PBS with 0.05% Tween-20) and incubated overnight at 4°C with primary antibody, washed, then again incubated for 1 hour with horseradish peroxidase or alkaline phosphatase conjugated secondary antibody. The bound antibody complexes were detected using the enhanced chemiluminescence (ECL) system or NBT/BCIP system respectively.

### Immunofluorescence of permeabilised cells

Indirect immunofluorescence staining of the cells was done by fixing the cells at specific time periods with paraformaldehyde (2%) and the cells were made permeable with glycine (0.1 M) and Triton X-100 (0.1%). All the chemicals and antibody dilutions were prepared in PBS (pH 7.2). After blocking with BSA (3%), the cells grown on cover slips were incubated with specific primary antibody for 2 h. For detection of proteins, secondary antibody raised against either mouse, goat or rabbit were used. These were fluorescence tagged either with Alexa Fluor 488 or 546. To analyze nuclear morphology, DAPI (5 mg/ml) (Sigma, USA) was added to the cover slip 15 min before secondary antibody washing. Thereafter, the cells were washed and mounted in 50% Glycerol in PBS. Fluorescence images were monitored using an Axioscope microscope (Carl Zeiss, Germany) equipped with epifluorescence and Axiocam camera system coupled with Axio Vision software (Carl Zeiss, Germany) or by Nikon 90i microscope (Nikon Instech Co. Ltd. Parale Mitsui Bldg. Japan) using Evolution QEi Digital Camera (Media Cybermatric, U.S.A.).

### MTT Assay

0.2×10^5^ cells were seeded in each well of a 24 well culture cluster. The samples were collected at required time point in triplicates. 10 µl of MTT (1 mg/ml) dye was added to each well and incubated at 37°C for 4 h in a humidified CO_2_ incubator. The precipitate formed was solubilised in 150 µl of the solubilisation buffer DMSO after throwing away the media and the absorbance was recorded when all samples were collected. The colored formazan product is stable at 4°C for several days. The absorbance was recorded at 570 nm.

### Assay of intracellular ROS in cell lines

Intracellular H_2_O_2_ production was detected by fluorescence of 2',7'-dichlorodihydrofluorescein diacetate, acetyl ester, H_2_DCFDA (10 µM) incubated under various conditions for 10 min in dark as previously reported [29].

### Morphological Analysis by Haematoxylin – Eosin Staining

Haematoxylin-Eosin (H-E) staining of cells was performed according to the procedure mentioned earlier [28].

### HA Staining

The cells seeded on cover slips were fixed with 2% paraformaldehyde in PBS for 15 min at room temperature and then washed with 0.1 M glycine for 5 min to quench excess aldehyde. Cells were then permeabilized using 0.1% Triton X-100 v/v for 1 min and the excess detergent washed off by PBS. The cells were incubated with commercially available Biotinylated HABP for 1 h, washed with PBS and then incubated with secondary antibody, Streptavidin conjugated with Alexa Fluor 430 (1∶300) for 1 h. To analyze nuclear morphology, DAPI was added to the cover slip 15 min before secondary antibody washing. Thereafter, cells were washed and mounted in PBS-glycerol and then observed under microscope.

### Statistical analyses

ImageJ software was used to analyze the band intensities of immunoblots and after normalization with loading controls the relative fold changes were calculated. All data were expressed as mean ± standard deviation (SD) of observations in triplicate (n = 3), unless mentioned otherwise. Single factor one-way statistical analysis of variance (ANOVA) or unpaired t-test was carried out using software whenever applicable. Difference at a level of *p<0.05 and **p<0.005 between groups were considered as statistically significant.

## Results

### Morphology of HepG2 and HepR21 cells remain unchanged without autophagic vacuolation and redox sensitivity

**Figure 1:**
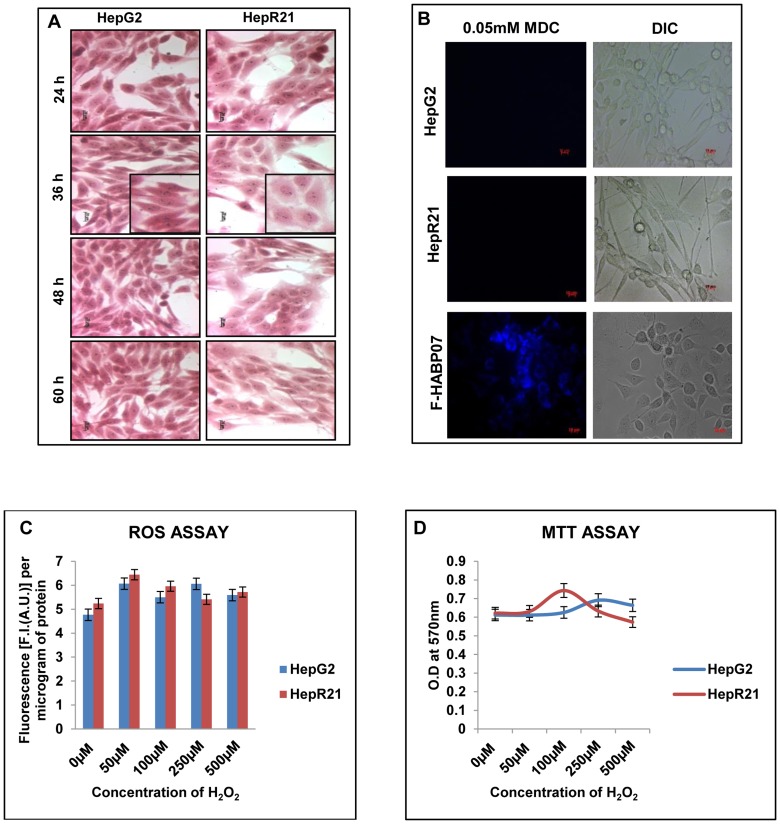
Morphology of HepG2 and HepR21 remains unaffected on progression of growth with both cell lines showing redox insensitivity. [A] ***Haematoxylin-Eosin Staining*** **—** Morphological analysis by H-E staining of HepG2 and HepR21 cells illustrated that HABP1 transformed HepG2 i.e. HepR21 although morphologically varied than its normal counterpart having a higher cell volume, with a more flattened spread out appearance showed no change in morphology as a function of time. Increased generation of vacuolated HepR21 cells upon progression of time point was not observed. A magnified view of both the cells for 36 h of growth has been shown. [B] ***MDC staining shows absence of autophagic vacuolation in both HepG2 and HepR21 cells*** **—** HepG2 and HepR21 cells were grown for different periods starting from 36 to 84 h and subjected to MDC (0.05 mM) staining as per the protocol mentioned in Methods. Subsequent fluorescence microscopy indicated no positive staining for autophagic vacuoles for any point of growth between 36 h to 84 h. MDC staining of F-HABP07 cells was taken as positive control. Scale bar represents 10μ. [C] ***HepG2 and HepR21 cells are both insensitive to external redox stimuli*** **—** ROS assay performed on HepG2 and HepR21 cells after prior treatment of cells with varying concentration of H_2_O_2_ (0 µM, 50 µM, 100 µM, 250 µM and 500 µM) for one hour indicated both HepG2 and HepR21 cells to be redox insensitive even on exposure to 500 µM of H_2_O_2_. [D] ***H_2_O_2_ treatment has no effect on survivability of HepG2 and HepR21 as evident from MTT Assay*** **—** HepG2 and HepR21 cells were grown in complete media till 48 h and then treated with the abovementioned concentrations of H_2_O_2_ for 1 h. Viability assay performed thereafter revealed that even 500 µM of H_2_O_2_ has no effect on the survivability of both the cell lines. The media was not changed at any point.

Overexpression of HABP1 in normal fibroblasts (F-HABP07) leads to ROS induced autophagic vacuolar manifestation during 36, 48 and 60 h of growth [28]. To test whether overexpression of HABP1 in hepatocarcinoma cell line, HepG2, leads to a similar effect on vacuole formation, cells were stained with H-E and MDC as described in materials and methods. In contrast to fibroblasts, stable overexpression of HABP1 in HepG2 cells, already containing high levels of endogenous antioxidants, showed no vacuolar manifestations. Rather H-E staining of both HepG2 and HepR21 showed retention of their respective morphology as a function of growth **(** **Figure 1A** **)**. This observation was further confirmed by staining the cells with the autofluorescent compound MDC which specifically stains autophagic vacuoles. Fluorescence microscopic analysis indicates no positive staining for autophagic vacuoles for any point of growth between 36 h to 84 h for either HepG2 or HepR21. However, F-HABP07 cells taken as a positive control showed autophagic vacuole as evident by robust MDC staining (**Figure 1B**). This was consistent with previous results showing unchanged intracellular ROS levels in HepG2 and HepR21 cells [32]. In order to determine whether external oxidant stimuli had any effect on intracellular ROS levels, HepR21 cells were treated with increasing concentration of H_2_O_2_. As controls, HepG2 cell were also subjected to identical treatment. In both parent and HABP1 overexpressed HepG2 cell lines, internal ROS remained unchanged upon external oxidant stimuli (**Figure 1C**). Consistently, there was no significant change in viability of either HepG2 or HepR21 cells upon treatment with H_2_O_2_ (**Figure 1D**).

### Autophagic marker MAP-LC3 and the tumor suppressor PTEN are downregulated in HepR21 cells as compared to HepG2 cells

**Figure 2:**
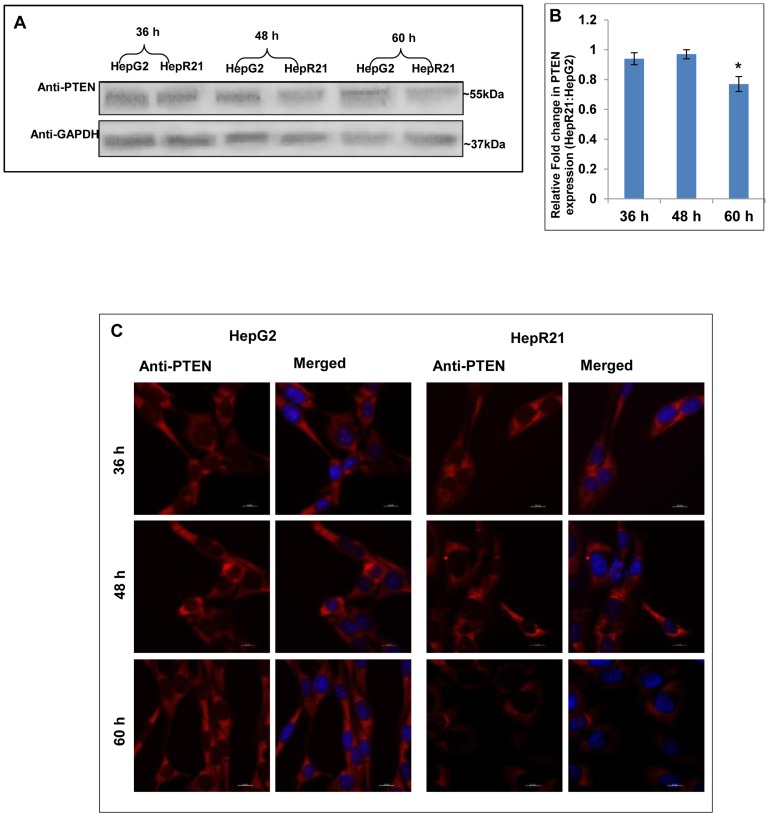
Downregulation of tumor suppressor PTEN upon HABP1 overexpression in HepG2. [A-B] ***Downregulation of expression of the autophagic modulator PTEN in HepR21*** **—** Autophagic modulator and also a known tumor suppressor PTEN was observed from immunoblots to have a significantly reduced expression (∼30%) in HepR21 cells compared to HepG2 after 60 h of growth, indicating a higher tumorigenicity of HepR21. Fold changes were calculated taking GAPDH as loading control, using ImageJ and expressed as mean ± standard deviation (SD) of observations in triplicate (n = 3). Statistical analysis of significance was done by Single factor one-way ANOVA (*p<0.05). [C] ***Immunocytochemical analysis corroborates immunoblot findings*** **—** Decreased expression of PTEN was also detected in HepR21 cells at 60 h of growth compared to that in HepG2 from cytochemical analysis.

Tumor suppressor PTEN has been shown to regulate autophagy in cancerous cells [37]. Given that HepR21 is significantly more tumorigenic than HepG2 cells [32] we measured changes in PTEN levels and autophagic marker MAP-LC3. To determine any difference in expression of tumor suppressor PTEN between HepG2 and HepR21 cells, PTEN levels were monitored by immunoblot assay at distinct growth points in these cells lines (**Figure 2A**). Interestingly it was found that PTEN levels were downregulated significantly in HepR21 cells at 60 h of growth (**Figure 2B**). The immunofluorescence assay corroborates the immunoblot analysis, indicating decreased PTEN levels in HepR21 cells grown for 60 h (**Figure 2C**).

**Figure 3:**
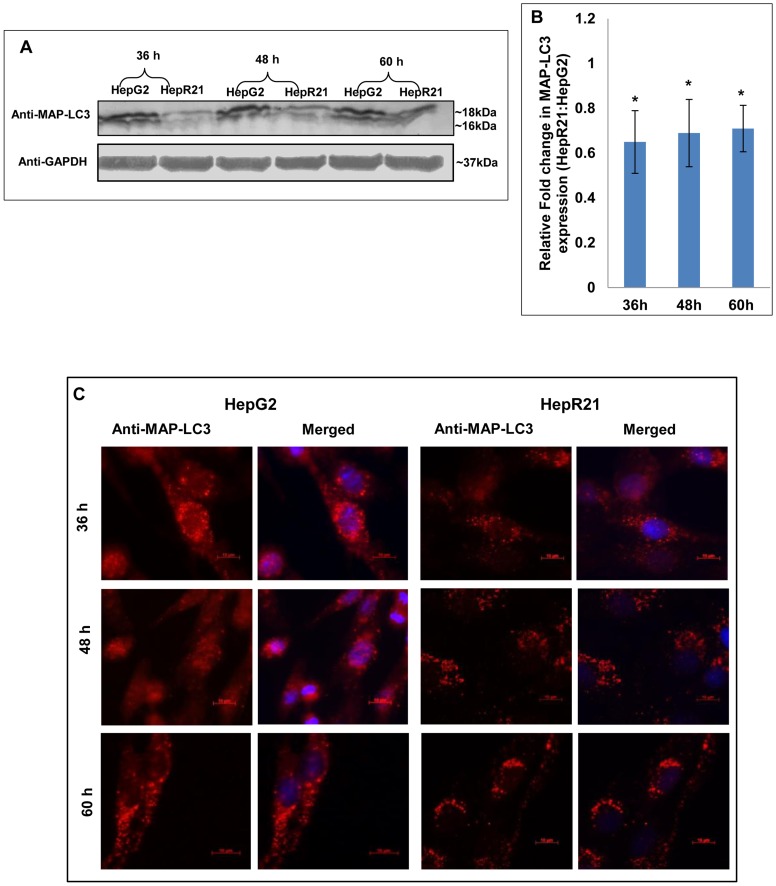
Downregulation of autophagic marker MAP-LC3 in HepR21. [A] ***HepR21 cells show decreased expression of MAP-LC3 in immunoblots*** **—** Immunoblotting of HepG2 and HepR21 cells grown for various time periods clearly depicts a downregulated expression of the autophagic marker MAP-LC3 in HepR21 cells. **[B]** ***Significant downregulation of MAP-LC3 expression in HepR21*** **—** Fold change analysis after normalization with the loading control GAPDH using ImageJ and further statistical analysis by ANOVA indicates significant downregulation of total MAP-LC3 in HepR21 cells compared to HepG2 cells for all the time periods. Fold changes are represented as mean ± SD (n = 3, *p<0.05) **[C]** ***Immunocytochemistry also illustrates downregulation and punctate staining of MAP-LC3 in HepR21*** **—** Immunocytochemical analysis done using the antibody for MAP-LC3 and subsequent reprobing with anti-rabbit Alexa Fluor 546 and DAPI revealed decreased expression of the autophagic marker MAP-LC3 with HABP1 overexpression in HepR21 as compared to the non-transformed HepG2 cells grown for 36, 48 and 60 h. The characteristic punctate staining of MAP-LC3 was obtained in both the cell lines.

In order to assess the expression profile of the autophagic marker, both HepG2 and HepR21 cells were grown for varied periods (36, 48 and 60 h) and processed for either immunoblotting or immunofluorescence analysis. Significant downregulation of MAP-LC3 in HepR21 cells was observed from immublotting and subsequent statistical analysis for all the growth periods (**Figure 3A and 3B**). Punctate staining, characteristic of MAP-LC3 was observed for both HepG2 and HepR21. HepG2 cells showed a higher expression of this protein than HepR21 as observed in the western blot. However, there seemed to be no augmentation in the expression levels with increased period of growth in either HepG2 or HepR21 cell lines (**Figure 3C**).

### Resistance of HepR21cells is greater than HepG2 cells to glutathione depletion

**Figure 4:**
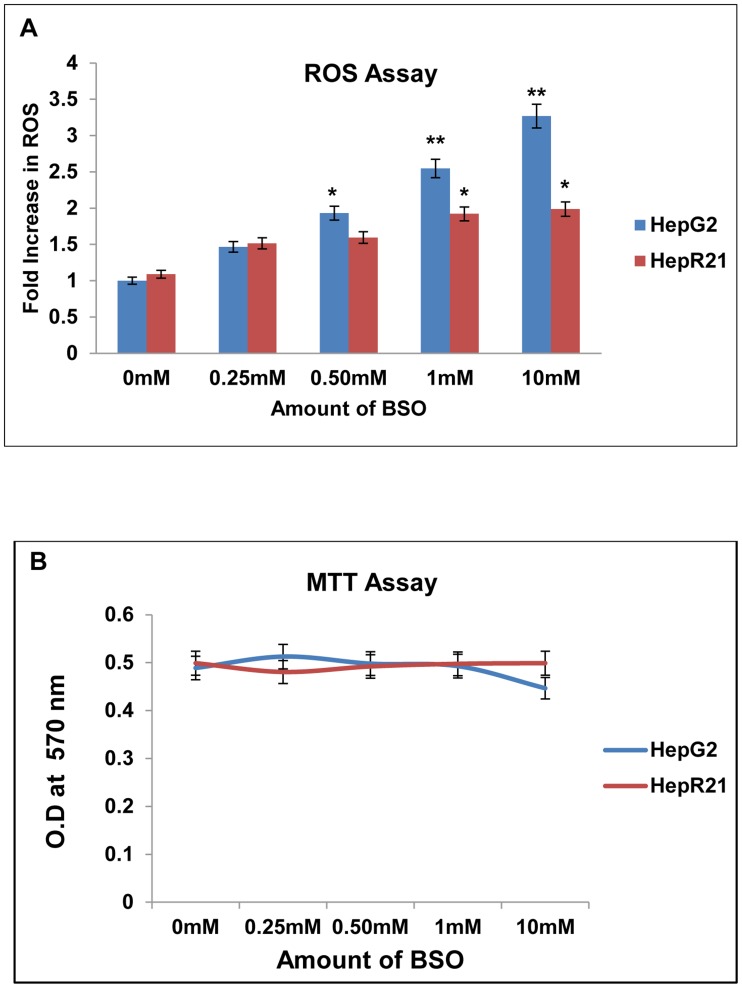
HepR21 cells have higher resistance to glutathione depletion compared to HepG2 cells. [A] ***Significant surge in reactive oxidant level in HepG2 with exposure to BSO in a concentration dependent manner*** **—** HepG2 and HepR21 cells were treated with increasing concentrations of BSO (0.25 mM, 0.50 mM, 1 mM and 10 mM) for 24 h. ROS assay of the treated and untreated cells was performed and the fold change in ROS of the treated cells was calculated against untreated HepG2 cells taken as control. Progressive increase in ROS levels with increase in concentration of BSO in HepG2 starting from 1.5 to 3.25 folds was observed. On the contrary, HepR21 cells showed negligible increase in ROS levels initially compared to untreated HepG2 cells upon exposure to lower concentrations of BSO. Unpaired t-test using GraphPad indicated significant increase in ROS in HepG2 cells from 0.50 mM BSO treatment compared to the untreated HepG2 cells. The surge in ROS in HepG2 cells was highly significant for treatment with 1 mM and 10 mM BSO. Compared to HepG2 cells, HepR21 cells showed only a slightly significant rise in ROS levels for 1 mM and 10 mM BSO with respect to (w.r.t.) untreated HepG2 cells. Difference at a level of *p<0.05 and **p<0.005 between groups were considered as statistically significant. [B] ***Survivability of both HepG2 and HepR21 cells remains unaffected*** **—** Cell survivability assay performed for the abovementioned treatments indicated no detrimental effect on both the cell lines.

Buthionine sulfoximine (BSO) is a synthetic amino acid which irreversibly inhibits gamma-glutamylcysteine synthetase, thereby depleting the Glutathione (GSH) level in cells. GSH is a metabolite that plays a critical role in protecting cells against oxidative stress and thus its depletion results in free radical generation [38]. HepR21 cells have already been reported to have higher levels (∼2 folds) of GSH, as compared to HepG2 [32]. Hence, the effect of inhibition of GSH on the redox profile as well as cell viability of HepG2 and HepR21 cells were examined upon treatment with increasing concentrations of BSO. Intracellular ROS measurements were carried out using the redox responsive dye H_2_DCFDA and the fold increase was calculated against untreated HepG2 cells. In HepG2 cells, intracellular ROS levels increased by 1.5 fold at 0.25 mM BSO concentration. Addition of higher concentrations of BSO led to further increase in intracellular ROS level (**Figure 4A**). In fact a direct correlation of ROS levels and added concentration of BSO was observed for HepG2 cells and the increase was found to be significant for 0.50 mM to 10 mM with the significance being very high for 1 mM (∼2.5 folds) and 10 mM of BSO treatment (∼3.2 folds). In HepR21 cells, negligible change in intracellular ROS levels was observed upon exposure to 0.25 mM BSO. However, in contrast to HepG2 cells, addition of higher concentration of BSO to HepR21 cells only resulted in a significant increase (∼2 folds) in intracellular ROS for 1 mM and 10 mM of BSO. ROS levels in untreated HepG2 cells were taken as control (**Figure 4A**). Although there was difference in the levels of ROS generation in HepG2 and HepR21 cells upon BSO addition, this was not significantly detrimental to cell viability (**Figure 4B**).

### HepR21 cells have a higher tolerance to nutrient deprivation in comparison to HepG2 cells

**Figure 5:**
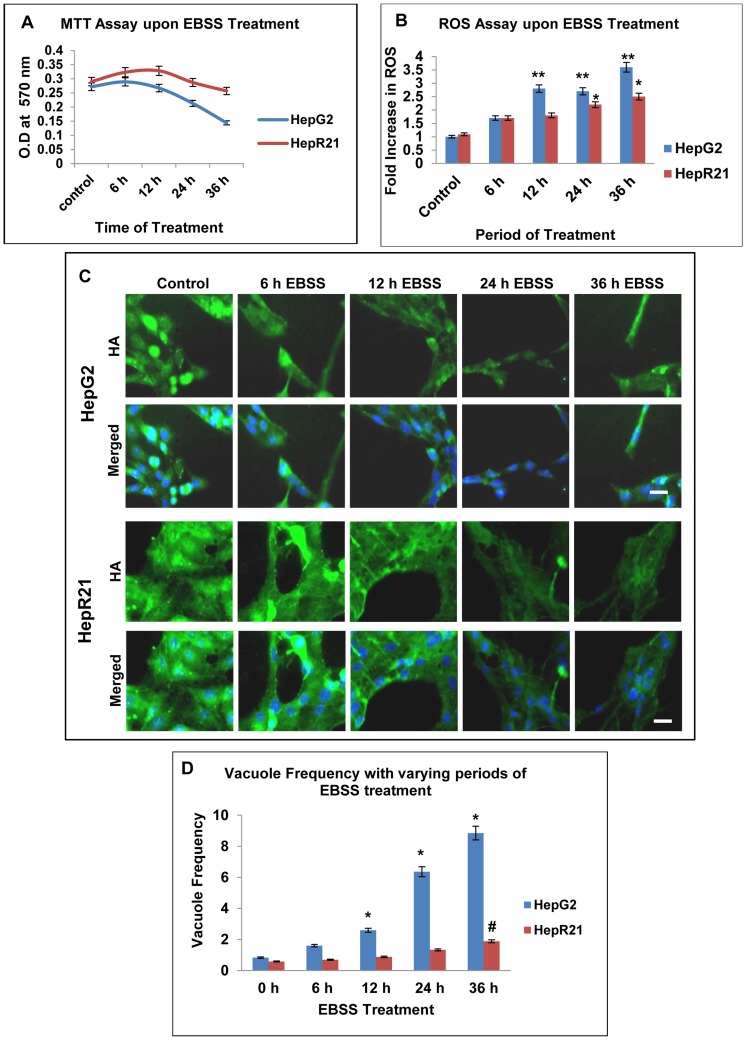
HepR21 cells have increased resilience to nutrient deprivation compared to HepG2 cells. [A] ***Cell survivability assay*** **—** Effect of nutrient starvation on HepG2 and HepR21 cells was studied by treatment with nutrient deficient media, EBSS for 6, 12, 24 and 36 h. A set of cells were kept as control. The survivability of HepR21 cells was found to remain unaffected while HepG2 cells were highly sensitive upon prolonged starvation with an overall 50% decrease in growth after 36 h of starvation. [B] ***Assay for ROS*** **—** ROS assay performed after the aforementioned treatments showed an increased ROS generation of 1.7 fold for 6 h to 3.6 fold for 36 h of nutrient starvation, found to be highly significant for 12 to 36 h of treatment for HepG2 (**). While significant increase in ROS of 2.2 to 2.5 fold in case of HepR21 cells (*) compared to untreated HepG2 cells was observed for 24 to36 h of nutrient starvation (*p<0.05 and **p<0.005, n = 3). [C] ***Decline in endogenous HA and HA cables with increased period of nutrient starvation*** **—** Untreated and nutrient deprived HepG2 and HepR21 cells were immunocytochemically stained with commercial biotinylated HABP, then subsequently with Streptavidin conjugated to Alexa Fluor 430 and DAPI. Decreased levels of HA and HA cables with increased nutrient starvation was observed, more prominently in HepG2 cells while decrease in HA cables in HepR21 was observed after prolonged treatment only. Scale bar represents 10μ. [D] ***Increased vacuole frequency upon amino acid deprivation in HepG2 compared to HepR21*** **—** Amino acid deprived media, EBSS, induced progressively increased generation of vacuoles in HepG2 with increasing period of nutrient deprivation (6, 12, 24 and 36 h) compared to untreated cells. Almost 10 fold increase in vacuole frequency in HepG2 cells was observed after 36 h of nutrient deprivation, while for the same treatment the HepR21 cells showed a mere 3 fold increase as compared to the untreated control cells. Statistical analysis using ANOVA with difference at a level of *p≤0.05 between groups considered as significant, revealed significant increase in vacuoles for 12, 24 and 36 h of nutrient starvation in HepG2 (*); while the increase was only significant after 36 h of EBSS treatment for HepR21, denoted as **#** in the figure.

Given that HepR21 cells have a greater tumorigenicity as well as resistance to ROS, we determined whether nutrient deprivation had any effect on HepR21 cells. HepG2 and HepR21 cells were treated with amino acid deficient media, EBSS for increasing periods of time. As control, cells were grown under the same condition in complete media. Difference in cell viability and redox sensitivity were determined. Cell survivability of HepR21 cells remained constant while, HepG2 cells were highly sensitive upon prolonged starvation with an overall 50% decrease in growth (**Figure 5A**). Internal ROS measurement indicated a highly significant fold increase in ROS production in HepG2 cells as early as 12 h (∼2.8 folds) of nutrient starvation. In contrast, level of ROS increase in HepR21 cells was only significant after prolonged starvation of 24 h (∼2.2 folds) and 36 h (∼2.5 folds) of nutrient starvation compared to the untreated HepG2 cells (**Figure 5B**).

Nutrient deprivation is known to generate elevated intracellular ROS levels [39]–[41]. Also known is that endogenous HA acts as a scavenger molecule for excess ROS [42]–[44]. Given that HepR21 cells upon nutrient starvation accumulate much lower ROS levels than HepG2, we checked the status of HA in the nutrient starved HepG2 and HepR21 cells. Decrease in levels of HA with increase in time period of nutrient starvation was evident from the immunocytochemical analysis of both control and nutrient starved HepG2 and HepR21 cells. Interestingly, the decline in HA level as consequence of nutrient deprivation was more prominent in HepG2 cells than HepR21 cells (**Figure 5C**). In HepR21 cells the commencement of decrease in HA cables coincided with increase in ROS generation upon prolonged nutrient starvation (**Figure 5B and 5C**).

Serum and nutrient starvation leads to ROS generation and elevated ROS levels result in upregulation of autophagic machinery. HepG2 cells have been reported to undergo autophagy on nutrient deprivation by the use of amino acid deficient medium, EBSS [35]. Consistently, we observed a proportional increase in number of vacuolated cells upon duration of nutrient deprivation in HepG2 cells. Incubation in nutrient deprived medium led to ∼10 fold increase in vacuole frequency after 36 h of starvation in HepG2 (**Figure 5D**). Interestingly, the accumulation of vacuoles in HepG2 also coincided with upsurge in ROS and decline in HA upon nutrient starvation (**Figure 5B and 5C**). In contrast, the number of vacuolated cells in HepR21 almost remained constant for the first 24 h after incubation with EBSS. Upon further incubation with EBSS the number of vacuolated cells tripled compared to untreated cells (**Figure 5D**).

### Nutrient starvation leads to increased expression of HABP1, MAP-LC3 and differential localization of tumor suppressor p14ARF in HepG2 unlike HepR21

**Figure 6:**
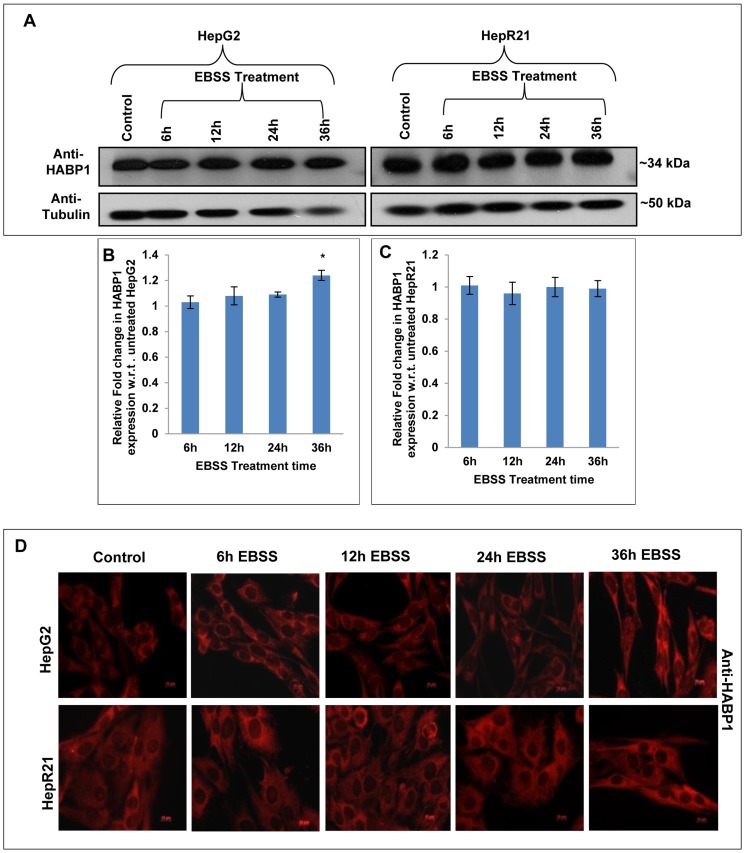
Prolonged nutrient deprivation leads to upregulated expression of HABP1 in HepG2 cells only. [A-C] ***Immunoblotting for HABP1*** **—** Whole cell lysates of HepG2 and HepR21 cells nutrient deprived for varied periods (6, 12, 24 and 36 h) along with untreated controls were immunoblotted with rabbit polyclonal anti-HABP1 (1∶1250) and anti- Tubulin (1∶5000). HABP1 expression levels were normalized against Tubulin and the fold change calculated with respect to untreated controls using ImageJ and further analyzed using ANOVA. A prominently increased expression of HABP1, after prolonged nutrient starvation of 36 h in HepG2 cells was detected (*p<0.05, n = 3). The expression of HABP1 remained unchanged for the said periods of nutrient starvation in HepR21 cells. [D] ***Elevated levels and nuclear translocation of HABP1 in HepG2 after 36 h of nutrient starvation*** **—** Immunocytochemistry also confirmed an increased HABP1 expression in HepG2 cells after 36 h of nutrient starvation. Translocation of a fraction of HABP1 to the nucleus was also observed for 36 h nutrient deprived HepG2 cells. As expected, HepR21 cells stably overexpressing HABP1 showed a higher expression of the protein compared to HepG2, but no further increase or nuclear translocation on increasing the period of nutrient starvation was perceived.

To determine changes in expression levels of HABP1/MAP-LC3/tumor suppressor p14ARF in HepR21 cells, tolerant to nutrient starvation; immunoblotting of EBSS treated and untreated HepG2 and HepR21 cell lysates were performed. Blots were normalized to the level of tubulin or GAPDH and fold change was calculated with respect to untreated controls. The expression level of HABP1 remained unchanged under nutrient deprived condition in HepR21 cells as compared to the untreated control cells. However, only in HepG2 cells, significant upregulation in HABP1 levels upon nutrient deprivation after 36 h was observed (**Figure 6A, 6B and 6C**). As observed in the immunoblots, immunocytochemical staining of HepR21 and HepG2 cells also corroborates the unchanged levels in HepR21 cells and the increased HABP1 expression after 36 h of nutrient starvation only in HepG2. In addition HepR21 cells had higher HABP1 levels in comparison to HepG2 cells as expected. Interestingly, a fraction of HABP1 in HepG2 cells was found in the nucleus upon prolonged nutrient starvation of 36 h (**Figure 6D**).

**Figure 7:**
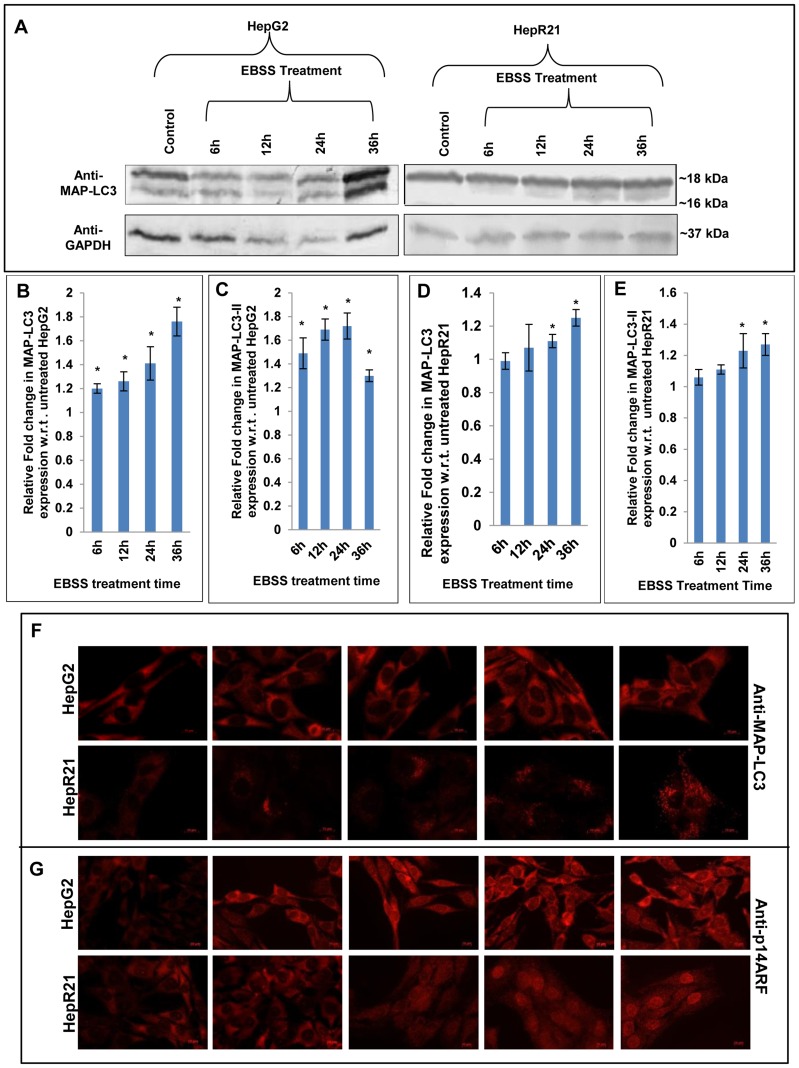
Upregulated MAP-LC3-II, differential expression and localization of tumor suppressor p14ARF in HepG2 unlike HepR21 upon nutrient deprivation. [A-E] ***Elevated levels of total MAP-LC3 and MAPLC3-II in HepG2 upon nutrient starvation*** **—** After the aforementioned treatment with EBSS, the HepG2 and HepR21 cell lysates were immunoblotted with MAP-LC3 (1∶1000) and GAPDH (1∶20,000) antibodies. The fold change in expression of MAP-LC3 was calculated after normalization with GAPDH expression by IMAGE J and subsequently analyzed using ANOVA and represented in the graph as mean ± SD. The analysis showed a progressively increased expression of total MAP-LC3 in HepG2 along with increased period of starvation, significant for all the treatments compared to the untreated HepG2 cells. The expression of MAP-LC3-II (16 kDa), the lipidated form of MAP-LC3, indicative of the amount of autophagy taking place, was also found to be significantly upregulated for the abovementioned exposure to EBSS in HepG2 cells. Statistical analysis revealed a significantly increased expression of total MAP-LC3 and MAP-LC3-II only after 24 h of nutrient starvation in HepR21 cells (*p<0.05, n = 3). [F] ***Cytochemical expression of autophagic marker MAP-LC3 upon nutrient starvation*** **—** As observed in the immunoblot, an augmented expression of the autophagic marker MAP-LC3 was observed in HepG2 cells by sequential probing with anti-MAP-LC3, anti-rabbit Alexa Fluor 546 and DAPI on fixed, treated and untreated cells. While the HepR21 cells showed the characteristic punctate staining throughout with only a slight rise after prolonged nutrient starvation. [G] ***Differential expression and localization of the tumor suppressor p14ARF observed for HepG2 and HepR21*** **—** A comparative immunocytochemical analysis of the expression of the tumor suppressor protein, p14ARF in HepG2 and HepR21 indicated an elevated expression with the increase in period of nutrient deprivation in both the cell lines, more prominently in HepG2. Interestingly, a prominent differential localization of the protein in the two cell lines was also noticed. While the protein was predominantly expressed in the cytoplasm in HepG2 cells, it was observed to be translocated to the nucleus progressively with nutrient deprivation in case of HepR21 cells.

Immunoblot analysis of autophagic marker, MAP-LC3; revealed nutrient deprivation leads to significant increase in both total MAP-LC3 expression and MAP-LC3-II expression (16 kDa) in EBSS treated HepG2 cells (**Figure 7A, 7B and 7C**). In addition we observed a linear correlation between its increased expression and time of starvation in this cell line. In contrast, the HepR21 cells showed only a slight increase in expression of total MAP-LC3 and MAP-LC3-II only after 24 h of nutrient starvation and beyond (**Figure 7A, 7D and 7E**) which coincided with comparatively increased vacuole frequency in HepR21 cells (**Figure 5D**). Immunofluorescence microscopy for MAP-LC3 also corroborates the above observation (**Figure 7F**).

Differential response of HepG2 and HepR21 upon nutrient starvation with respect to expression of autophagic markers led us to examine the expression pattern of p14ARF, the human counterpart of mouse p19ARF. p14ARF is a known tumor suppressor and a regulator of p53 which has been implicated in autophagic regulation via its short mitochondrial form smARF [23]. smARF is produced by internal initiation of translation which is being stabilized by HABP1/p32 [22]. Thus, levels of p14ARF were examined in nutrient deprived HepG2 and HepR21 cells. Fluorescence microscopy revealed that the nutrient starved HepG2 cells have prominently higher expression of this protein and p14ARF is localized mainly in the cytoplasm, mostly in the nuclear periphery (**Figure 7G**). Interestingly the expression pattern of p14ARF in HepR21 cells was found to be distinct from the HepG2 cells. In HepR21 cells, p14ARF translocation to the nucleus was observed from 12 h of nutrient starvation, and it increased with the starvation period suggesting that differential localization of p14ARF might have a role in the differential behavior of the two cell lines upon nutrient starvation.

### HAS inhibition by 4-MU treatment in HepR21 leads to decreased HA levels and as a consequence increased ROS, upregulation of MAP-LC3 and tumor suppressor PTEN

**Figure 8:**
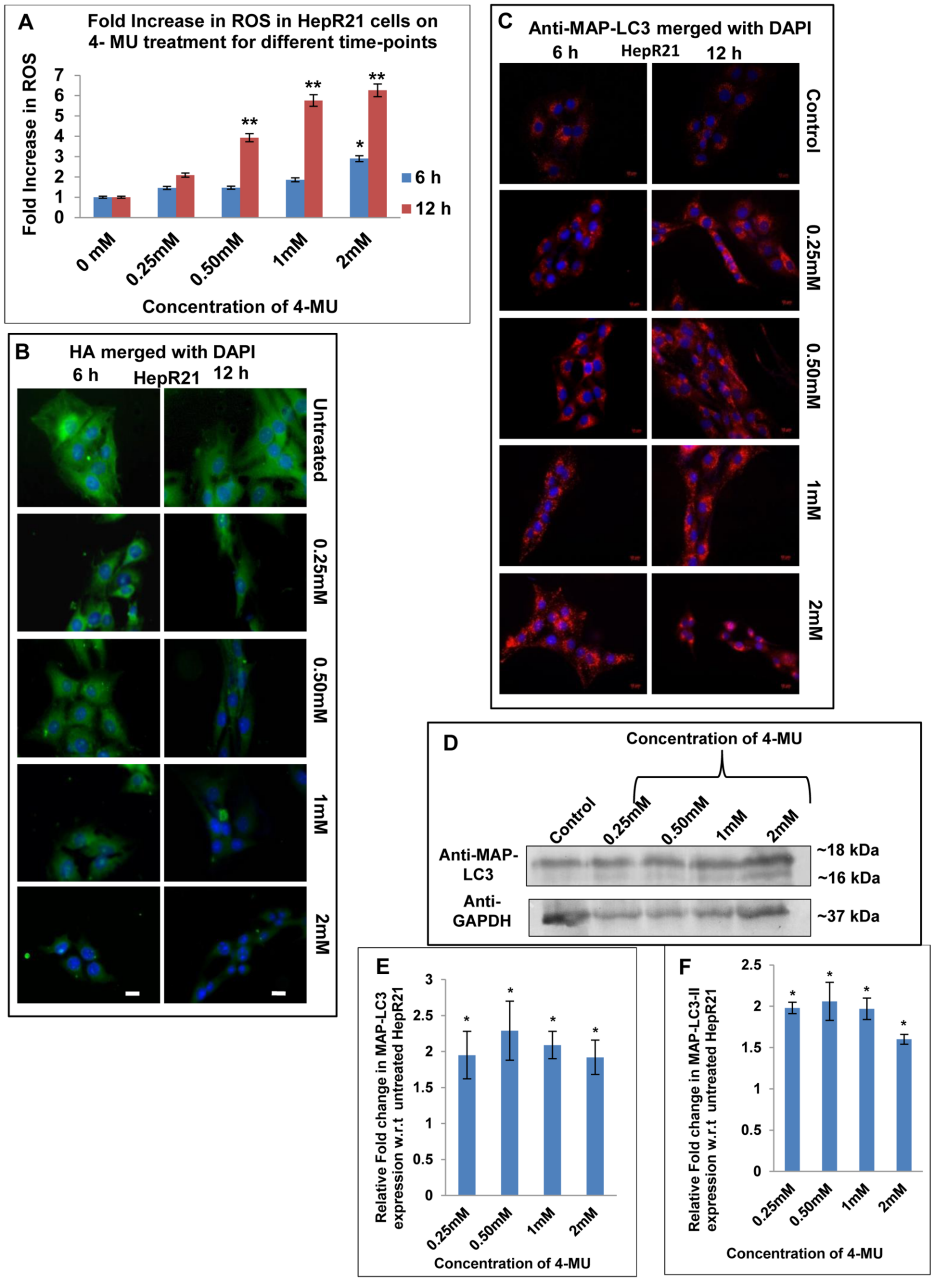
HAS inhibition in HepR21 leads to ROS surge, decline in HA level and upregulated MAP-LC3 levels. [A] ***Inhibition of HA synthesis led to increased generation of ROS*** **—** HepR21 cells subjected to treatment with different concentrations of HA synthetase inhibitor, 4-MU and untreated cells were assayed for ROS. 4-MU treatment resulted in excess generation of ROS as a function of time and concentration. The assay showed a ROS fold increase of 1.5 to 3 with increase in 4-MU concentration from 0.25–2 mM upon treatment for 6 h, with the increase being significant (*) for 2 mM of 4-MU treatment. Statistical analysis indicated highly significant increase in ROS from 0.50 (∼4 folds) to 2 mM (∼6.3 folds) for 12 h of 4-MU treated cells compared to untreated HepR21 cells, denoted by **. [B] ***Decline in HA levels*** **—** HepR21 cells earlier reported to have higher HA pool were subjected to the aforementioned treatments with 4-MU and immunocytochemically analyzed for HA. Immunocytochemical analysis using commercial Biotinylated HABP and then reprobing with streptavidin cy3 and DAPI, illustrated decreasing level of HA with increasing concentration of 4-MU and the change to be more drastic for 12 h of treatment. The cells can be observed as individual entities, which were beforehand intricately linked with HA cables. Scale bar represents 10μ. [C] ***Immunocytochemistry shows upregulation of MAP-LC3 upon inhibition of HA synthesis in HepR21 cells*** **—** Immunocytochemical detection of the autophagic marker MAP-LC3 revealed an increased expression after 6 h of 2 mM 4-MU treatment while it showed a prominent upregulation of the protein in the 12 h treated cells which were previously observed to generate excess ROS. [D-F] ***Immunoblotting validates cytochemical analysis of upregulated MAP-LC3 levels*** **–** Immunoblots for MAP-LC3 of 4-MU treated (12 h) and untreated samples and further analysis as described previously revealed significant (*) upregulation of total MAP-LC3 and MAP-LC3-II for all the 4-MU treated samples compared to untreated HepR21 cells. This observation signifies induction of autophagy to be concomitant with HA depletion and ROS surge.

It has already been reported that the survivability of HepR21 cells, is compromised upon exposure to the HAS inhibitor 4-MU which depletes the cellular UDP-glucuronic acid, downregulating HAS2 and HAS3 [33]. Given that HA levels are elevated in HepR21 cells in comparison to HepG2 and remained elevated even upon nutrient starvation (**Figure 5C**), we determined the consequences of HA depletion in HepR21 cells on ROS levels, autophagic markers and tumor suppressor PTEN. HepR21 cells were treated with increasing 4-MU concentrations for either 6 or 12 h. The presence of oxidants in the untreated controls and treated cells was tested using the redox-responsive fluorescent dye H_2_DCFDA as described in Methods. The fold increase in ROS was calculated against the control values and the results were plotted as fluorescence per microgram of protein. It was observed that 4-MU treatment significantly increased generation of ROS both as a function of time and concentration (**Figure 8A**). The 4-MU treated and untreated cells were also processed for immunostaining for HA as per the protocol mentioned in the Methods section. Fluorescence microscopic analysis indicated that depletion of HA levels and loss of HA cables were dependent on 4-MU concentration as well as the length of treatment (**Figure 8B**). The decline in HA coincided with the increase in ROS generation upon 4-MU treatment. The cells can be observed as individual entities, which were beforehand intricately linked with HA cables.

To determine the levels of autophagy, immunocytochemical analysis of HepR21 cells for the expression status of the autophagic marker MAP-LC3, was measured after 4-MU treatment. We observed a prominent upregulation of MAP-LC3 in the 12 h treated cells (**Figure 8C**) coinciding with increased generation of ROS (**Figure 8A**). Punctate staining of MAP-LC3 can be observed around the nuclear periphery which increased with increasing concentration of 4-MU after either 6 h or 12 h of treatment. The levels of 4-MU needed to increase MAP-LC3 puncta was greater (2 mM) when cells were treated for 6 h (**Figure 8C**). Though only about 50 percent cells survived after 2 mM of 4-MU treatment for 12 h, the cells were seen to be shrunk and rounded up, but, highly dense accumulation of MAP-LC3 in the nuclear periphery was observed. The immunofluorescence data was further validated by immunoblotting of 12 h treated HepR21 cells along with untreated control, revealing significant increase in both total MAP-LC3 and MAP-LC3-II levels for all the aforementioned treatments compared to the untreated cells (**Figure 8D, 8E and 8F**).

**Figure 9:**
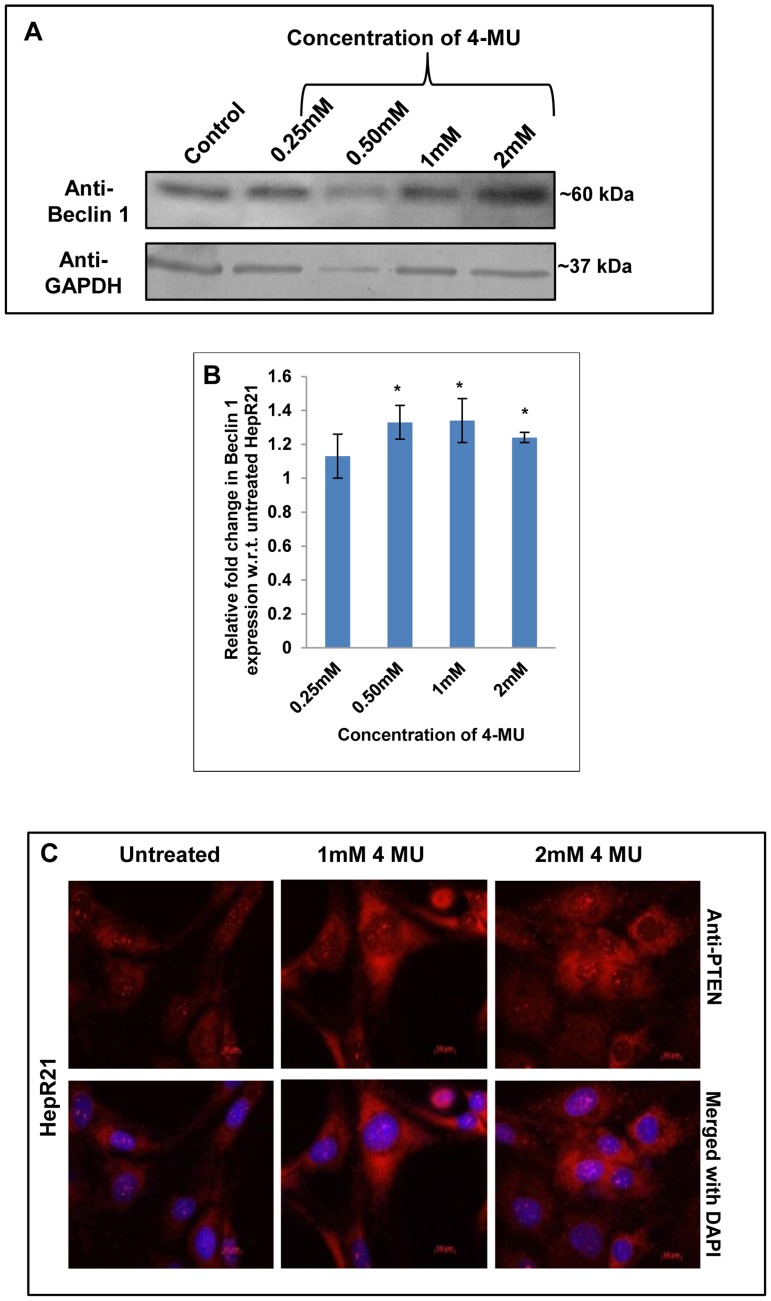
HA depletion leads to induction of autophagy and upregulation of tumor suppressors. [A-B] ***Upregulated Beclin 1 in HepR21 upon decline in HA*** **—** Simultaneous immunoblotting of the 4-MU treated and untreated cells using anti-Beclin 1 and anti-GAPDH also revealed a significant (*) upregulation of the other autophagy marker and tumor suppressor, Beclin 1 upon 4-MU treatment in HepR21 cells. [C] ***Immunocytochemical analysis for PTEN*** **—** HepR21 cells having increased tumor potency showed an upregulation of the autophagy modulator and tumor suppressor when subjected to 4-MU treatment suggesting a decline in tumor potency upon induction of autophagy.

To determine whether there were changes in tumor suppressor levels, Beclin 1 and PTEN levels were measured. Immunoblotting of the 4-MU treated and untreated cells also revealed a substantial upregulation of the other autophagy marker and tumor suppressor, Beclin 1 upon 4-MU treatment (**Figure 9A and 9B**). Consistent with Beclin 1 upregulation, immunocytochemical analysis of HepR21 cells after 4-MU treatment also led to elevated expression of the autophagic modulator and tumor suppressor PTEN (**Figure 9C**). Given that there was an increase in tumor suppressors and autophagy upon 4-MU treatment, it is highly probable that HepR21 cells treated with 4-MU will have reduced tumorigenicity.

## Discussion

Autophagy acts as the alternative cell death pathway which might come into play when apoptosis is impaired. This property can be utilized against cancerous cells. Though HA is known to be elevated in tumors nothing much is known yet to relate it with autophagy. In the present study we conclude that, ROS mediated autophagy in hepatocarcinoma cell line can be regulated with the scavenging activity of the higher level of endogenous HA, indirectly controlling the tumor potency. Conclusions are made using the ROS insensitive HepG2 stable transfectant, HepR21 overexpressing HABP1 [32] and containing increased endogenous HA and HA cables. These characteristics of HepR21 has been utilized to correlate between HABP1 mediated HA upregulation with autophagy and tumor potency by nutrient starvation and HA depletion with HAS inhibitor. Firstly, this cell line was found to be resistant to oxidative stress without occurrence of autophagic vacuoles upon HABP1 overexpression. Secondly, the tumor suppressor, PTEN and the autophagic marker MAP-LC3 were also found to be downregulated in HepR21. Even glutathione depletion was unable to cause as significant an increase in ROS level in HepR21 as in the case of its parent cell line HepG2. Thirdly, HepR21 cells were more resistant than its parental cells towards nutrient starvation, an autophagy inducer. These cells showed increased vacuolation and MAP-LC3 upregulation only after prolonged starvation. In contrast, significantly increased ROS and vacuolation in HepG2 with augmented expression of the autophagic marker MAP-LC3 was observed upon nutrient starvation of even a shorter period. Surprisingly, HepR21 cells, having high levels of endogenous HA and demonstrated to be ROS insensitive earlier; generated excess ROS immediately upon depletion in HA level and HA cables brought forward by HAS inhibitor, 4-MU. This observation was accompanied with an increased expression of autophagic marker MAP-LC3-II and upregulation of tumor suppressors Beclin 1 and PTEN expression.

The cellular redox potential is an important determinant of cell function. In several cases the supra-basal levels of ROS, generated in response to low doses of ionizing radiation or chemicals in the environment have been observed to bring about even beneficial cellular responses leading to cellular growth [45], [46]. Development of stress condition and growth inhibition upon overexpression of HABP1, whether be it transient in COS cells [47] or constitutive overexpression in fibroblast cells (F111) [29], [48] have been reported earlier. Excess generation of ROS in the HABP1 transformed F111 cells has been found to be responsible for autophagic vacuolation [28] as a manifestation of stress. On the contrary, HABP1 overexpression in HepG2 cells (HepR21) results in no excess ROS generation, cellular stress and apoptosis rather it induced enhanced cell growth and proliferation over longer periods. These cells had altered morphology, showing higher cell volume, having a more spread out appearance than HepG2 cells suggesting, HepR21 cells to be more adherent to each other. Although, the nuclear cytoplasmic ratio of HepR21, has been found to be comparable with that of its normal counterpart [32]. When HepR21 cells were monitored for the occurrence of any vacuolated structures no increased incidence of vacuolated structures were found from 24 to 60 h of growth, as observed in the other HABP1 transformed fibroblast cell line indicating that this cell line behaves differently than the other HABP1 transformed cell lines. Confirmation for no incidence of increased autophagic vacuolation upon constitutive overexpression of HABP1 was done using the autophagy specific dye MDC. Moreover, immunoblotting and immunocytochemical analysis clearly depicted a downregulation of the autophagic marker MAP-LC3 in the HABP1 transformed HepR21 cells as compared to HepG2 cells. In both the cell lines, the punctate staining as characteristic of MAP-LC3 and localization pattern was found to be similar.

Apart from absence of excess ROS generation due to overexpression of HABP1 in HepR21, both cell lines HepG2 and the transformed HepR21 were found insensitive to ROS toxicity with 500 µM of H_2_O_2_ treatment. This observation can again be attributed to the innate property of the hepatocarcinoma cell lines containing an elevated level of variety of antioxidant enzymes [31] to fight oxidative assault.

The tumor suppressor PTEN is the most commonly mutated gene in most cancers including hepatocellular carcinoma. Overexpression of PTEN inhibits HepG2 cell growth via cell cycle arrest and also inhibits migration and invasion without inducing apoptosis [49]. Its expression has been shown to be highly downregulated in HepG2 cells among several other HCC cell lines as compared to human liver immortal cell line L02 [50]. The drastic reduction in PTEN expression in HepR21 could explain the enhanced cell growth as observed in our earlier report [32]. Moreover, it is reported that PTEN deficiency elevates the level of phosphorylated Akt leading to increased tumorigenesis in hepatocytes [51]. This is also applicable to our experimental system, HepR21 having higher level of activated Akt, indicating more tumorigenic capability [32].

Higher intracellular GSH level reportedly play a critical role in protecting cells against oxidative stress and induces cell proliferation [52]. Glutathione Assay conducted earlier on the HepG2 and HepR21 cells has indicated about 2 fold increase in levels of GSH in HepR21 cells which has been implicated as one of the reasons for enhanced cellular proliferation in this cell line [32]. However, the differential response of the two cell lines towards glutathione depletion by BSO to ROS generation suggests that the increased glutathione cannot be solely responsible for ROS insensitivity in HepR21. Probably, the higher pool of HA in HepR21 cells [32] and its property of being a ROS scavenger [42]–[44] might have a role to play here. It is important to mention about the interesting reports suggesting the protective effects of GSH upon HA degradation due to the thiol antioxidative effect of GSH [53] and the increased levels of GSH involved with inhibition of hyaluronidase [54] resulting in increased endogenous levels of polymeric HA. Hence, the augmented GSH levels in HepR21 cells might have a role in elevating the amount of endogenous HA. Thus, the changed behavior and higher tolerance of the HepR21 cells might be attributed to the elevated endogenous levels of HA and CD44. The increased HA level is also an indicator for higher tumorigenic capability of the cell line [32]. The hyaladherin CD44 is reported to be involved with promotion of tumorigenesis [4], [55] by the activation of survival pathways upon binding with HA.

Several reports have implicated serum and nutrient starvation in the generation of ROS which mediates autophagy [39]–[41] and HA's role as a scavenger of ROS [42]–[44] has also been well documented. Drastically diminished HA level in the HepG2 cells compared to HepR21 cells was concomitant with the surge in ROS in HepG2. HepR21 cells also revealed negligible increase in the frequency of vacuoles and cellular survivability upon nutrient starvation as compared to the parent cell line HepG2. Thus, it can be postulated that the increased HA levels in the HepR21 cells are responsible for its protection from induction of autophagy through starvation. Compounds that activate Protein Kinase C isoforms (PKCs), like phorbol esters, can increase HA biosynthesis in mammalian cells. TGFβ and PDGF, which are found to be increased in cancerous cells also play a significant role in elevating the activity of HAS [56], [57]. The multifunctional chaperone protein p32/HABP1 has been shown to interact with all PKC isoforms, regulating them and it has also a stimulatory effect on one of the isoforms PKCδ, whose expression can be increased 2 fold in presence of HABP1 [58], [59]. Thus, overexpression of HABP1 in HepR21 might have a role in increased activity of HAS, resulting in increased HA levels and cable formation.

HABP1 has been implicated to be upregulated during stress situations such as, in chryptorchid rats during spermatogenic arrest or in the instance of apoptosis induction by Cisplatin in HeLa cells or in the hypoxic and nutrient deprived portions of tumors [60], [61]. Nutrient deprivation of 36 h probably acting as a stress inducer in HepG2 cells led to the upregulation and nuclear translocation of the protein; whereas, HepR21 already had higher endogenous level of HABP1. Ten folds increase in vacuole frequency might be a ramification of the increased stress in the nutrient deprived HepG2 cells and the upsurge in expression of MAP-LC3 can be correlated to the increased generation of vacuoles. On the contrary, slight elevated levels of MAP-LC3 after 24 h to 36 h of nutrient starvation in HepR21 resonated with the increased vacuole frequency for that starvation period. All these observations again indicated the stably HABP1 overexpressing HepR21 cells to be more resilient towards nutrient starvation than its parent cell line HepG2. Differential localization of p14ARF in the two cell lines was detected. HepG2 cells showed a linear increase in expression of p14ARF with period of nutrient starvation and its cytoplasmic localization around the nuclear periphery was observed. On the contrary, p14ARF gradually translocated to the nucleus as a function of period of nutrient starvation in HepR21 cells. The tumor suppressor p14ARF acts as an inhibitor of MDM2 by sequestering it in the nucleolus, preventing its translocation to cytoplasm, resulting in p53 stabilization and activation [62]–[64]. The short mitochondrial form of p19ARF, smARF, had been shown to be associated with the induction of autophagy upon mitochondrial translocation after being stabilized by physical interaction with HABP1/p32 [22], [23]. Thus its presence in the cytoplasm and elevated expression might be responsible for the increased vacuole generation and increased levels of MAP-LC3 in HepG2. Nutrient deprivation in HepR21 cells led to differential behavior of HepR21 than its normal counterpart. These cells showed no autophagic induction till 24 h of nutrient starvation, but might lead to p53 activation and apoptosis due to the nuclear translocation of p14ARF.

Overexpression of HABP1, an enigmatic member of the hyaladherin family leads to dissimilar effects in different cell lines. This might be due to its differential subcellular localization in various cells and thus a myriad ligand binding capacity resulting in altered cellular signaling. Upon overexpression in normal fibroblast (F-HABP07) this protein primarily accumulates in the mitochondria leading to excess generation of ROS [29]. Recent report suggests ROS mediated HA depletion, depolymerization and autophagic vacuole formation prior to apoptosis induction in HABP1 overexpressing normal fibroblasts, which can be reverted back with polymeric HA supplementation [28]. In the ROS insensitive cell line HepR21, HABP1 level remains high and localizes on the cell surface, inducing cellular proliferation, higher endogenous levels of HA and generation of intercellular HA cables in HepR21 [32]. This upsurge in HA level and cable formation alters the cellular signaling making the cells resilient towards ROS assault and nutrient deprivation. Thus, inhibiting HA synthesis in HepR21 cells by 4-MU, the HAS inhibitor, depleted the HA pool and cables, concomitant with the ROS generation. The survivability of HepR21 cells gets highly affected upon HA depletion by 4-MU treatment [33], which is not the case upon exposure to either BSO or EBSS. Increased punctate staining of the autophagy marker MAP-LC3 along with significant upsurge in MAP-LC3-II and Beclin 1 expression with escalated concentration of 4-MU used was noted, which was concurrent with the surge in ROS generation. It is of huge importance that only prolonged exposure to nutrient deprived media resulted in significant fold increase in ROS for HepR21 cells without affecting cell survivability. Highly significant ROS fold increase can be achieved upon exposure to low concentrations of 4-MU. This is indicative of the fact that depletion of HA caused significant high levels of ROS generation (∼3.9 folds) and autophagy induction, which is reflected in the augmented expression of MAP-LC3-II and Beclin 1. The expression of the tumor suppressor, PTEN, which was earlier observed to be downregulated in HepR21 cells, also got augmented when the autophagy markers were upregulated, implying decreased tumor potency upon autophagy induction. 4-MU treated HepR21 cells are also reported to have upregulated expression of another tumor suppressor p53 [33] which also corroborates our findings.

Thus, we propose the elevated HA level and HA cables in HepR21 protects this cell line from generating excess ROS and autophagic vacuolation which can be reversed with HAS inhibitor suggesting HA mediated signaling may reflect its characteristic for being more tumorigenic.
